# A flexible plasma-treated silver-nanowire electrode for organic light-emitting devices

**DOI:** 10.1038/s41598-017-16721-7

**Published:** 2017-11-28

**Authors:** Jun Li, Ye Tao, Shufen Chen, Huiying Li, Ping Chen, Meng-zhu Wei, Hu Wang, Kun Li, Marco Mazzeo, Yu Duan

**Affiliations:** 1 State Key Laboratory on Integrated Optoelectronics, College of Electronic Science and Engineering, Jilin University, Jilin, 130012 China; 2 Key Laboratory for Organic Electronics and Information Displays & Institute of Advanced Materials (IAM), Jiangsu Nation Synergetic Innovation Center for Advanced Materials (SICAM), Nanjing University of Posts & Telecommunications, 9Wenyuan Road, Nanjing, 210023 China; 3 College of Computer Science and Technology, Jilin University, Changchun, 130012 China; 4 Istituto di Nanotecnologia, CNR-Nanotec, c/o Campus Ecotekne via Monteroni, Lecce, 73100 Italy

## Abstract

Silver nanowires (AgNWs) are a promising candidate to replace indium tin oxide (ITO) as transparent electrode material. However, the loose contact at the junction of the AgNWs and residual surfactant polyvinylpyrrolidone (PVP) increase the sheet resistance of the AgNWs. In this paper, an argon (Ar) plasma treatment method is applied to pristine AgNWs to remove the PVP layer and enhance the contact between AgNWs. By adjusting the processing time, we obtained AgNWs with a sheet resistance of 7.2Ω/□ and a transmittance of 78% at 550 nm. To reduce the surface roughness of the AgNWs, a peel-off process was used to transfer the AgNWs to a flexible NOA63 substrate. Then, an OLED was fabricated with the plasma-treated AgNWs electrode as anode. The highest brightness (27000 cd/m^2^) and current efficiency (11.8 cd/A) was achieved with a 30 nm thick light emitting layer of tris-(8-hydroxyquinoline) aluminum doped with 1% 10-(2-benzothiazolyl)-2,3,6,7-tetrahydro-1,1,7,7-tetramethyl-1H,5 H,11H-(1)-benzopyropyrano(6,7-8-I,j)quinolizin-11-one. Compared to thermal annealing, the plasma-treated AgNW film has a lower sheet resistance, a shorter processing time, and a better hole-injection. Our results indicate that plasma treatment is an effective and efficient method to enhance the conductivity of AgNW films, and the plasma-treated AgNW electrode is suitable to manufacture flexible organic optoelectronic devices.

## Introduction

Flexible conductive electrodes play an important role in many optoelectronic devices, such as organic light-emitting diode (OLED)^1,2^, organic solar cells^3,4^, and touch screens^5^. Indium tin oxide (ITO) is the most common anode used for these applications. However, the price of ITO is increasing because of the relative rarity of indium and the high cost deposition. In addition, the brittle nature of ITO also restricts its utility in flexible devices^6^. To solve these problems, metal grids^7,8^, silver nanowires^9–16^, copper nanowires^17,18^, graphene^19,20^, carbon nanotubes^21,22^, and conductive polymers^23,24^ have been investigated as compelling alternatives to ITO. Among them, Silver nanowire (AgNW) with their excellent electrical, optical, and mechanical properties, is considered as one of the most promising materials to apply for flexible optoelectronic devices^25,26^. Silver nanowire films are usually prepared using solution-based processes, including spin-coating, drop-casting, rod-coating, and spray-coating. During these processes, the connection between crossed AgNWs is mainly determined by gravity, van der Waal forces between them, and capillary forces from solvent evaporation^27^. This can lead to loose contact between crossed AgNWs and produce a large contact resistance. In addition, the surfactant coating of polyvinylpyrrolidone (PVP) may be left on the surface of AgNWs, which further increases the sheet resistance of the film^28,29^. To reduce the sheet resistance, methods like high-temperature thermal annealing, applying extra pressure, washing with acetone, laser nanowelding, and adding other conductive materials, have been investigated. Among these, high-temperature thermal annealing requires a relatively long time to decompose PVP and fuse the Ag nanowires together^30^. Acetone washing uses ethanol to remove PVP on the surface of the Ag nanowires. However, the need for repeated stirring and centrifugation make it a complex procedure^28^. Mechanical pressure can reduce the contact resistance between the Ag nanowires, for example, by applying a pressure of more than 10 MPa. However, it requires rinsing in water and ethanol to remove PVP on the AgNWs’ surface^31^. Laser nanowelding is a fast process, which utilizes the enhanced surface plasmon resonance at the junction between neighboring AgNWs. Hence, it can only be used at the NW junction^32–34^. Some groups have added other conductive materials to enhance the conductivity of AgNWs. These include Au coating^35,36^, Ag nanoparticles^37^, carbon nanotubes^38^, and PEDOT:PSS^39^, which require an extra process. Some groups tried to use low temperature plasma to remove the PVP layer on the surface of AgNWs. Zhu Siwei *et al*. enhanced the conductivity of ultralong AgNWs by a room-temperature plasma treatment and obtained a high figure-of-merit (FOM) of 471^40^. Dae-Gon Kim *et al*. combine the plasma-treated AgNWs with a colorless polyimide and fabricate a high sensitivity touch sensor with it. Here, we use the plasma-treated AgNWs as anode to fabricate an OLED^41^. Compared to the touch sensor, the electrode of OLED requires lower sheet resistance and higher work function to get a good hole-injection. And the surface roughness needs to be smaller than the thickness of the buffer layer to avoid short circuit. In our paper, we selected a relatively high density of AgNWs to achieve good hole-injection. In same area, AgNWs with higher density have more AgNWs junctions, thus the AgNWs welding plays more important role in the decrease of sheet resistance. Besides, the work function can also increase by the plasma treatment. Another problem with AgNWs is their large surface roughness, which can easily penetrate the soft organic layer of a laminated device and produce short circuit. Some researchers tried to combine AgNWs with high adhesion transparent polymers^40,42–44^. Sanggil Nam *et al*. embedded AgNWs into NOA63 and Ki-Hun Ok *et al*.^45^ used a colorless polyimide to reduce the surface roughness. By these inverted process, the AgNWs were wrapped by the polymer, so the surface roughness of the hybrid films could be reduced to a very small value.

In this paper, we employ a low energy plasma process to the pristine AgNWs films. The simple plasma treatment can clearly decrease the sheet resistance, and it hardly affects the transmittance of the AgNWs. By adjusting the processing time, we obtained AgNWs with a σ_DC_/σ_Op_ of 204, which is four times the value of pristine AgNWs. We then spin-coated NOA63 on the plasma-treated AgNWs and peeled it off after curing with an ultraviolet lamp. After the plasma treatment, the surface of the AgNWs can become rugged. The inverted process can avoid the negative effect of plasma and get a low surface roughness. Then we fabricated an OLED device using such plasma-treated AgNWs as anode. To investigate the superiority of the plasma-treated AgNWs, we also fabricated a contrasted OLED with annealed AgNWs as anode. The better hole-injection indicates that our plasma-treated electrode is a good candidate for improvement of flexible OLED.

## Methods

### Preparation of the AgNWs electrode

**Figure 1:**
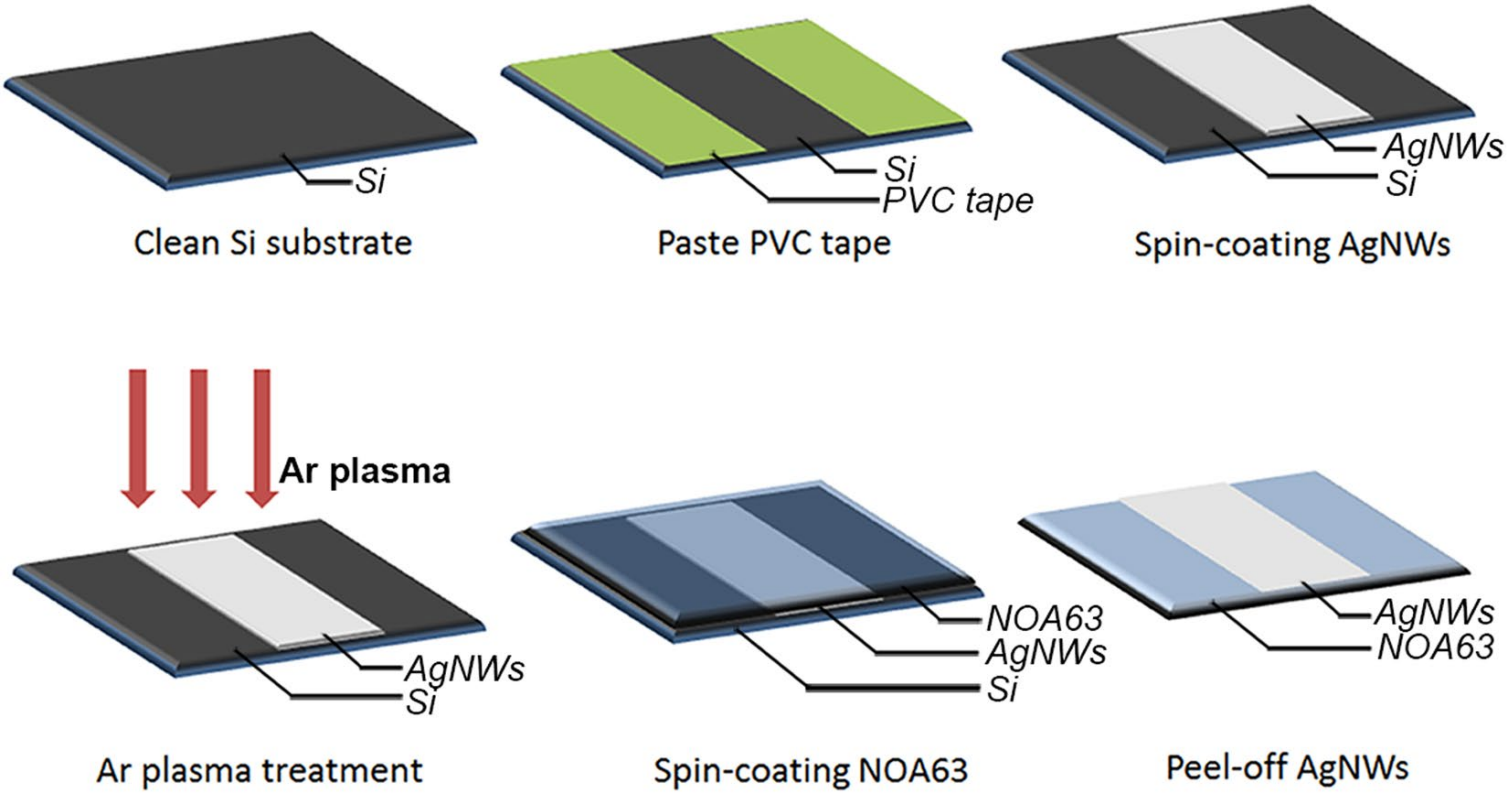
The fabrication process of the plasma-treated AgNWs electrode.

The AgNWs were purchased from JCNANO, with the average dimensions of 90 nm × 20 um. The photopolymer NOA63 was purchased from Norland Products. The plasma treatment was conducted using a plasma cleaner (PDC-001-HP) from Harrick Plasma. We diluted the AgNWs suspension concentration down to 5 mg/ml. The choice to select a AgNW suspension concentration of 5 mg/ml is the result of our optimization of the electrical and optical properties based on our previous work. The fabrication process of the plasma-treated AgNW electrode is illustrated in Fig. 1. The A silicon (Si) substrate (2.5 cm × 2.5 cm) was cleaned sequentially with acetone, ethanol, and deionized water. A PVC tape was used to define the area on the substrate. Before the coating process, the AgNWs were shaken for 5 minutes, followed by resting (30 minutes) to obtain a uniform dispersion without AgNW clusters. Then the AgNW suspension was spin-coated onto the Si substrate at 8000 rpm for 30 s. Subsequently, plasma treatment (45 W) was performed on the AgNW film. To avoid the oxidation of the AgNWs, we choose Ar to generate the plasma. Then NOA63 was spin-coated on the AgNWs film at firstly 400 rpm for 15 s, followed by 800 rpm for 15 s. Furthermore, ultraviolet light treatment was applied at a wavelength of 370 nm and 300 W, for 4 min. After the curing of NOA63, we peeled off the NOA63 from the Si substrate. The plasma-treated AgNWs were embedded in NOA63 and removed from the Si substrate.

### Fabrication of the OLED device

An OLED was fabricated using the plasma-treated AgNW electrode. The plasma treated AgNWs were used as anode, a 5 nm MoO_3_ was used as buffer layer, a 40 nm TAPC was used as the hole injection layer, a 30 nm tris-(8-hydroxyquinoline) aluminum (Alq3) doped with 1% 10-(2-benzothiazolyl)-2,3,6,7-tetrahydro-1,1,7,7-tetramethyl-1H,5 H,11H-(1)-benzopyropyrano(6,7-8-I,j)quinolizin-11-one (C545T) was used as the active layer, a 20 nm Alq3 layer was used as electron transport layer, and a 0.5 nm Liq and 100 nm Al film served as cathode.

### Characterization Methods

The sheet resistance was measured with a four-probe ST-21 system. The transmittance spectra were obtained with a UV3600 (SHIMADZU). SEM was conducted on a JSM-7500F field-emission scanning electron microscope. The surface roughness was measured using an atomic force microscope (AFM) (Dimension Icon, Bruker Corporation) in tapping mode. An Agilent B2902A source meter and a Minolta luminance meter LS-110 were simultaneously used to measure the current density, voltage, luminance (J–V–L) characteristics of the flexible OLED. The electroluminescence (EL) spectra were recorded with a Spectroscan PR655 spectrometer.

## Results

### Variation of the sheet resistance through plasma treatment

**Figure 2:**
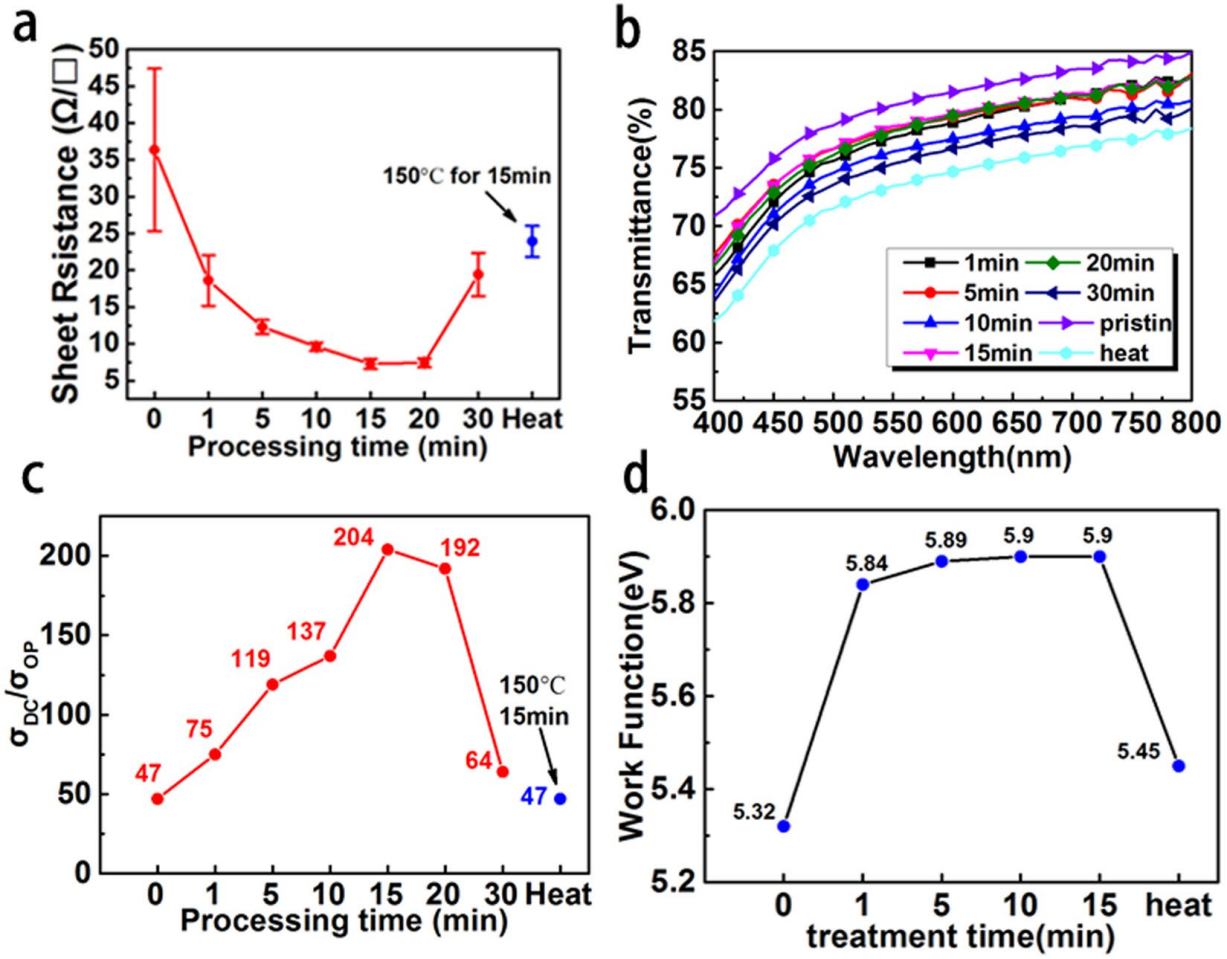
(**a**) Sheet resistances of the AgNWs for various plasma treatment times; (**b**) Transmittances of the AgNWs film for various plasma treatment times; (**c**) Ratios of σ_DC_/σ_Op_ for 550 nm with different plasma treatment times; (**d**) The work function of AgNWs with different treatment time.

The sheet resistance of the AgNWs film decreases significantly after plasma treatment. Figure 2(a) shows how the sheet resistance of the AgNWs changes with increasing processing time. The sheet resistance of the AgNWs gradually decreased when the processing time was within 15 min, and it became stable when processing time was between 15 min and 20 min. It then quickly increased after 20 min treatment. The smallest of sheet resistance was 7.2Ω/□ obtained after 15 min. All plasma-treated AgNWs films in Fig. 2(a) have a lower sheet resistance than the heated films, which were put into an oven at 150 °C for 15 min. Coskun^30^ *et al*. showed that the sheet resistance of AgNWs can decrease after thermal annealing. However, a long annealing time (200 min) is needed to achieve the optimal result. Our studies show that plasma treatment is a faster and more effective method than thermal annealing.

**Figure 3:**
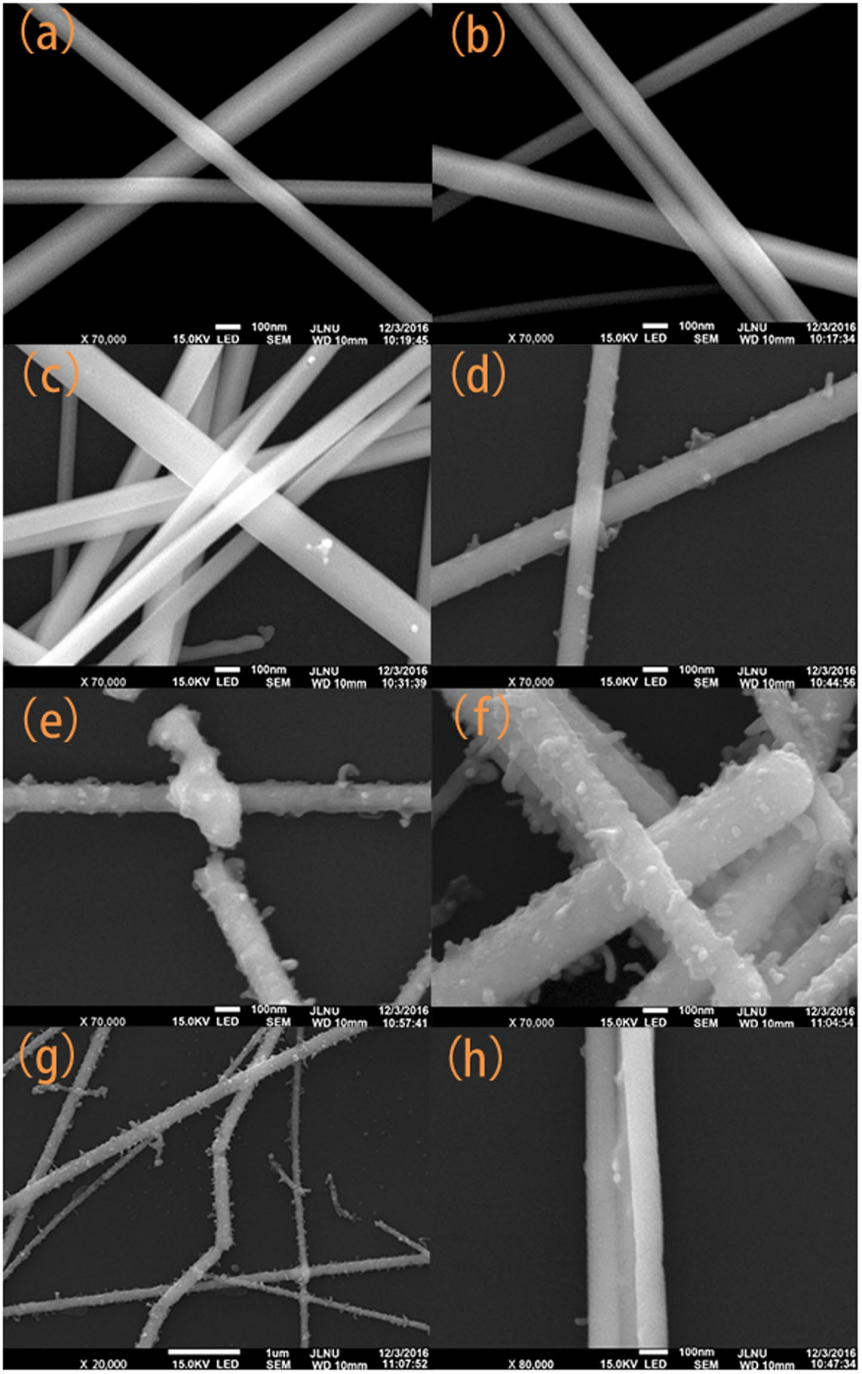
SEM images of AgNWs with different processing times. (**a**) Pristine AgNWs; (**b**) 1 min plasma treatment; (**c**) 5 min plasma treatment; (**d**) 15 min plasma treatment; (**e**) 20 min plasma treatment; (**f**) 30 min plasma treatment; (**g**) discontinuous points for 30 min plasma treatment; (**h**) fused AgNWs for 15 min plasma treatment.

Figure 2(b) shows the transmittance changes of the AgNWs for different processing times. The pristine AgNWs show the highest transmittance (80.4%) at wavelength of 550 nm. After plasma treatment, the transmittance of the AgNWs is slightly reduced, but it is still higher than the heated sample. The transmittance of AgNWs is mainly between 75.4% and 78.6% at 550 nm for increasing time from 1 min to 30 min. Compared to plasma process, the transparency drop of the AgNWs with thermal annealing process is more. It is because during the thermal annealing, the AgNW will spread at the junction and substrate^46^. During the plasma process, the thermal effect only happened on the surface of AgNWs. And the overall temperature is lower than thermal annealing. That leads to the higher transparency of plasma process.

The AgNWs after plasma treatment reduced the sheet resistance, while they increase the transmittance very little. To assess the overall effect of the plasma, we calculated the value of σ_DC_/σ_Op_, which is defined by Equation (1)^47^. This ratio has previously been used by other workers as a figure-of-merit for conductive thin films.

1 $${\rm{T}}(\lambda )={(1+\frac{188.5}{{R}_{sh}}\frac{{\sigma }_{op}(\lambda )}{{\sigma }_{DC}})}^{-2}$$

Here, T(λ) and R_sh_ are the transmittance and sheet resistance of the conductive thin film respectively. σ_op_(λ) is the optical conductivity and σ_DC_ is the DC conductivity of the film. Figure 2(c) shows the value of σ_DC_/σ_Op_ at 550 nm with different treatment times. The maximum σ_DC_/σ_Op_ value of 204 was obtained after 15 min plasma treatment. The value for the heated AgNWs is as low as the pristine AgNWs because of the inferior transmittance. This suggests that the AgNW film with 15 min plasma treatment has the best properties for a transparent electrode.

Figure 2(d) shows the work function of AgNWs with different treatment time. The result shows 1 min plasma treatment can effectively improve the wok function from 5.32ev to 5.84ev. Without plasma treatment, a thin layer of PVP is residual on the silver nanowire surface. This thin dielectric polymer can create interface dipoles, induce a vacuum-level shift and modify the work function of the AgNWs. After 1 min treatment, the most of the PVP is removed and the work function quickly enhanced. After 10 min treatment the work function is no longer changed, which means the PVP layer has completely removed from AgNWs surface. This result is consistent with the conclusion from sheet resistance change and SEM image.

SEM images of the AgNWs for different processing times are shown in Fig. 3. We can see that the morphology of the AgNWs starts to change after 5 min, see in Fig. 3(c). In Fig. 3(b), the morphology of AgNWs shows no discernible difference with Fig. 3(a). With increasing processing time in Fig. 3(c)–(f), more and more small grains appeared on the surface of the AgNWs. In Fig. 3(h), we can see two adjacent AgNWs fused together, which can decrease the contact resistance of the AgNWs network. For longer processing time, the melting of the AgNWs increased and it started to show some discontinuous points. In Fig. 3(g), we can see many discontinuous points in one single nanowire. As a result, the sheet resistance of the 30 min plasma treated AgNWs increased rapidly. In addition, the diameters of the AgNWs lie in a small range around the average length of 90 ${\rm{\mu }}$m^3^, and AgNWs with a smaller diameter are more likely to break during the plasma treatment.

### Surface roughness and flexibility of the flexible electrode

**Figure 4:**
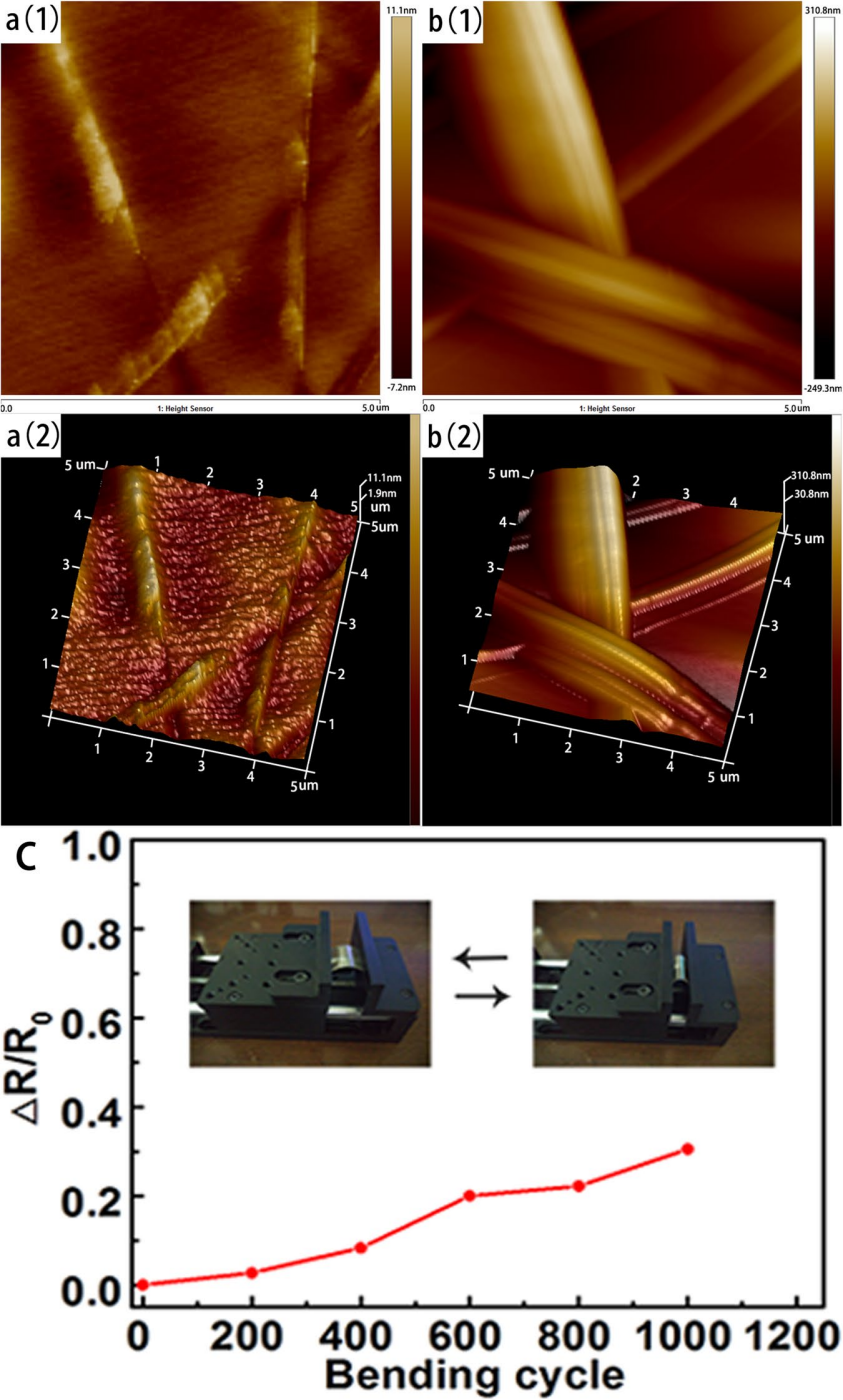
Surface roughness of AgNW films: a(1), a(2) AgNWs on NOA63; b(1), b(2) AgNWs on the Si substrate; c Sheet resistance change during the bending test.

Figure 4 shows the surface roughness of the AgNWs before and after the peel-off process. The root mean square (RMS) of the AgNWs on the Si substrate is 59.9 nm. After the peel-off process, the RMS of AgNWs on NOA63 decreased to 1.58 nm. In Fig. 4, we can see the AgNWs are embedded into the NOA63, and they produced a very small roughness. This low surface roughness is very important for OLED device because it reduces the possibility of short circuit. It is worth mentioning that the small grains on the AgNWs’ surface in Fig. 3 didn’t increase the surface roughness. It’s due to the inverted peel-off process, which transferred the AgNWs on the surface of the initial substrate to the bottom of the NOA63. Thus, the small grains are fully embedded into the NOA63 and it won’t influence the surface roughness of the electrode. Figure 4c shows the variation of sheet resistance during the bending test. The bending tests were performed by bending the films repeatedly (radius of curvature = 5 mm). After 1000 bending cycles, the increment of the sheet resistance was only 30% of the initial value. The low surface roughness and high flexibility means our electrode is suitable for OLEDs.

### Characteristics of the OLED

**Figure 5:**
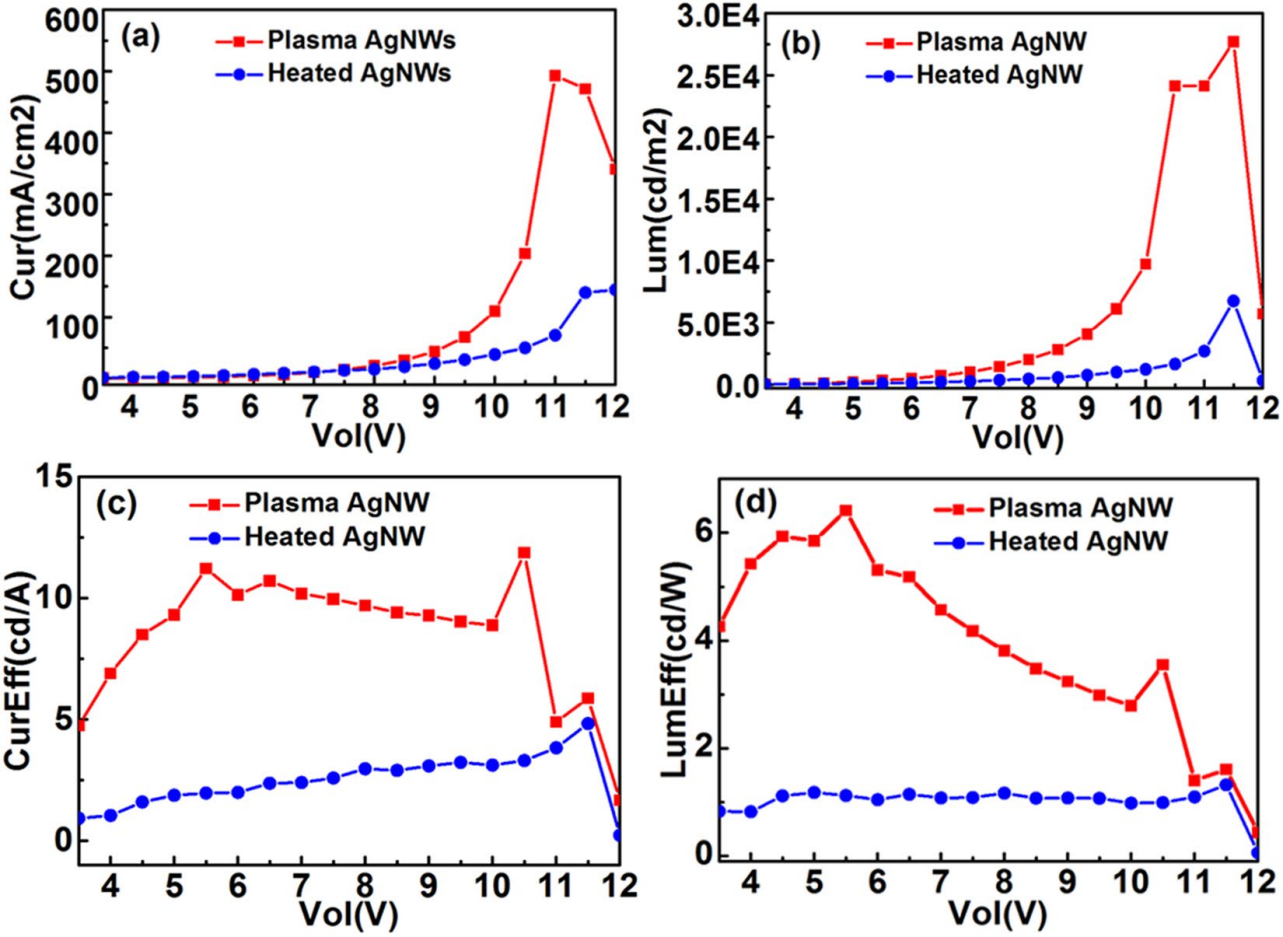
The J–V–L characteristics of OLED with plasma-treated and heated AgNWs as anode. (**a**) Current density-voltage curve; (**b**) luminance-voltage curve; (**c**) current efficiency-voltage curve; (**d**) luminous efficiency-voltage curve.

Figure 5 shows the J–V–L characteristics of the OLED for the plasma-treated and the heated AgNWs as anode. We can see in Fig. 5(a) that the current density for the OLED with plasma-treated AgNWs is 492.9 mA/cm^2^, which is much higher than the OLED with heated AgNWs. This is because both the sheet resistance and work function of the AgNWs increases after plasma treatment. The work function of AgNWs quickly increased in 1 min. And for 15 min plasma processing time, the work function achieved an increase of 0.58 eV. The results show that the plasma-treated AgNWs can provide good hole-injection in OLEDs. The luminance-voltage curve in Fig. 5(b) shows a similar variation tendency as Fig. 5(a). The structure of these two OLEDs is identical except for the anode. Hence, the better hole-injection of the plasma-treated AgNWs directly produces a higher luminance. In Fig. 5(c) and (d), the current efficiency and luminous efficiency of the OLED with plasma-treated AgNWs is much higher than the heated one. The host material Alq_3_ in this structure is an electron transport material, and a higher electron-current is facilitated in the light emitting layer. In other words, the enhancement of hole-current can significantly improve both the current efficiency and luminous efficiency. The maximum luminance and current efficiency of the OLED with plasma-treated AgNWs are 27000 cd/m^2^ and 11.8 cd/A, respectively. The improved hole-injection characteristic of the plasma-treated AgNW film makes it very suitable for OLED fabrication.

## Discussion

We also discussed the reaction that occurs during the plasma treatment. The SEM images show many small grains for plasma treatments of more than 15 min. We suspect the small grains stem from the thermal effect of the plasma. Because the plasma in this study is produced by a RF power in low pressure. It means in the plasma system, the heavy particles (gas molecules and ions) are essentially at ambient temperature (~0.025 eV), while the electrons have enough kinetic energy (several eV) to break covalent bonds^48^. The thermal effect of plasma is due to the impact of the high-energy electrons and the heat accumulating at the AgNWs surface. When the high energy electrons collide with the Ag atom, part of their kinetic energy turns into the internal energy of the AgNWs. With increasing processing time, the internal energy of the AgNWs and the temperature of the AgNWs surface increases. As a result, the AgNWs started to melt. When the plasma treatment was over, the melted part of the AgNWs can recrystallize. If the melting process occurred in the crossed or adjacent part of the AgNWs, the AgNWs can be fused more closely during the recrystallization process. The morphology of AgNWs shows no discernible change for 1 min treatment. However, the sheet resistance of the AgNWs changed from 36.35 Ω/□ to 18.6 Ω/□. We attributed this phenomenon to the etching effect of the plasma. The high energy and very active plasma particles hit the AgNWs, break the chemical bonds in PVP, and remove PVP from the AgNWs surface.

In summary, we used Ar plasma treatment and a peel-off process to prepare a flexible AgNW electrode. The sheet resistance of the AgNWs can be significantly reduced with the plasma, accompanied by little loss in transmittance. This is because the high-energy plasma can remove PVP from the surface of the AgNWs and fuse the AgNWs together where nanowires touch. After the peel-off process, the AgNWs were embedded into the NOA63 substrate, and the surface roughness was reduced significantly. A bending test was then conducted for the plasma-treated AgNW electrode. The test shows that this electrode can maintain its low sheet resistance after 1000 bending cycles. To prove the applicability of this electrode to build optoelectronic devices, we fabricated an OLED with plasma-treated AgNWs as anode. Compared to the optimized heated (150 °C for 15 min) AgNWs, the plasma-treated AgNWs exhibit better hole-injection. Thus, the OLED with plasma-treated AgNWs produced both higher luminance and higher current efficiency. Our experiment firstly demonstrated that plasma treatment is an effective and efficient method to enhance the conductivity of AgNWs in the solution process. The plasma-treated AgNW electrode may be used to improve flexible optoelectronic devices in the future.

## Electronic supplementary material

Supporting Information
