# Supplementary material for: A flexible plasma-treated silver-nanowire electrode for organic light-emitting devices
Source: Sci Rep. 2017 Nov 28;7:16468. doi: 10.1038/s41598-017-16721-7 (PMC5705724; doi:10.1038/s41598-017-16721-7)
Supplement: Supplementary file 1 — Supporting Information [file 41598_2017_16721_MOESM1_ESM.pdf]

A flexible plasma-treated silver-nanowire electrode for organic light-emitting devices

Jun Li[1], Ye Tao[1], Shufen Chen[2], Huiying Li[3], Ping Chen[1], Meng-zhu Wei[1,2], Hu Wang[1], Kun Li[1], Marco Mazzeo[4], Yu Duan[1,2]*

[1] State Key Laboratory on Integrated Optoelectronics, College of Electronic Science and Engineering, Jilin University, Jilin 130012, China

[2] Key Laboratory for Organic Electronics and Information Displays & Institute of Advanced Materials (IAM), Jiangsu Nation Synergetic Innovation Center for Advanced Materials (SICAM), Nanjing University of Posts & Telecommunications, 9Wenyuan Road, Nanjing 210023, China

[3] College of Computer Science and Technology, Jilin University, Changchun, 130012, China

[4] Istituto di Nanotecnologia, CNR-Nanotec, c/o Campus Ecotekne via Monteroni, Lecce 73100, Italy

Supporting Information

Figure S01

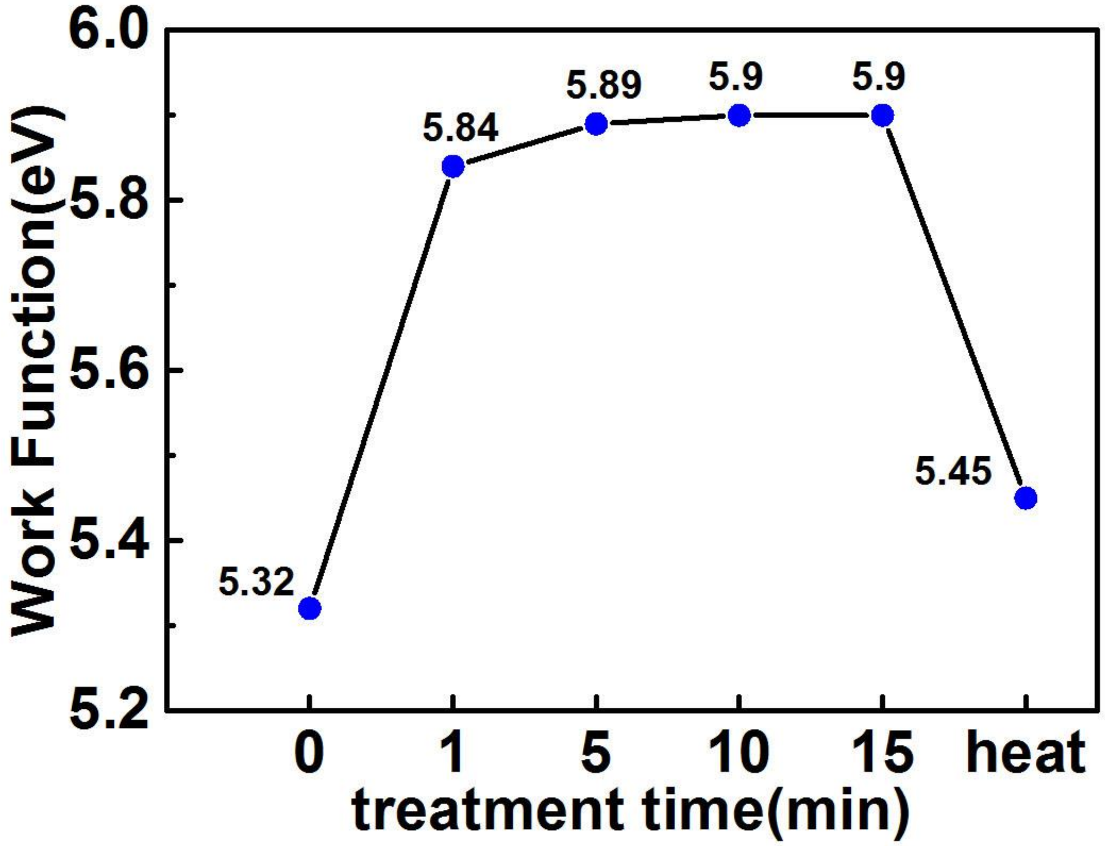

Figure S01. The work function of AgNWs with different treatment time.

Figure S02

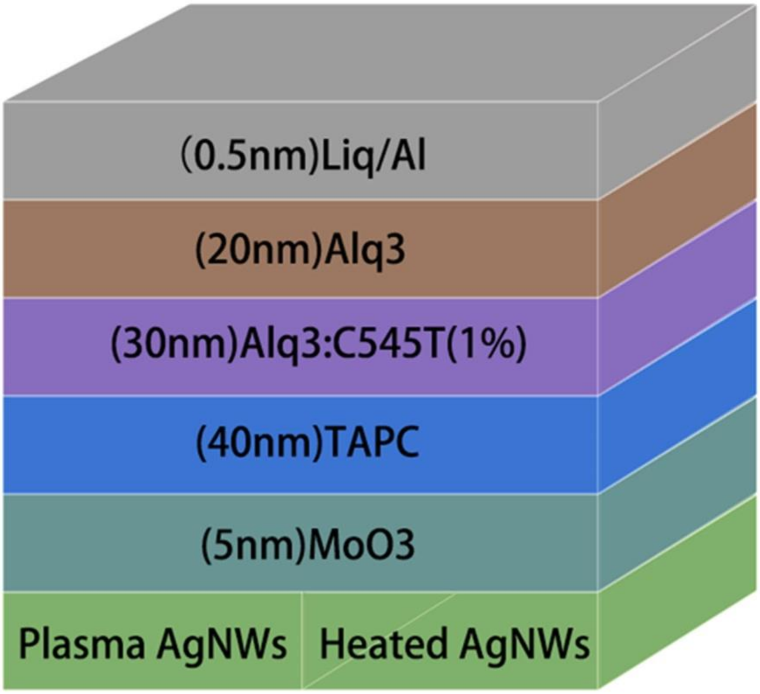

Figure S02. The structure of OLED.
